# Are early social communication skills a harbinger for language development in infants later diagnosed autistic?—A longitudinal study using a standardized social communication assessment

**DOI:** 10.3389/fcomm.2022.977724

**Published:** 2022-10-18

**Authors:** Shruthi Ravi, Allison Bradshaw, Hervé Abdi, Shoba Sreenath Meera, Julia Parish-Morris, Lisa Yankowitz, Sarah Paterson, Stephen R. Dager, Catherine A. Burrows, Chad Chappell, Tanya St.John, Annette M. Estes, Joseph Piven, Meghan R. Swanson, IBIS Network

**Affiliations:** 1 Department of Psychology, The University of Texas at Dallas, Richardson, TX, United States; 2 Department of Speech Pathology & Audiology, National Institute of Mental Health and Neurosciences, Bangalore, India; 3 Center for Autism Research, Children’s Hospital of Philadelphia, Philadelphia, PA, United States; 4 Boston Children’s Hospital, Harvard Medical School, Boston, MA, United States; 5 The James S. McDonnell Foundation, St. Louis, MO, United States; 6 Department of Radiology, University of Washington, Seattle, WA, United States; 7 Department of Pediatrics, University of Minnesota, Minneapolis, MN, United States; 8 Department of Psychiatry, University of North Carolina, Chapel Hill, NC, United States

**Keywords:** autism, language, social communication, longitudinal, infancy

## Abstract

The early emergence of social communication challenges and their impact on language in infants later diagnosed with autism has sparked many early intervention programs that target social communication skills. While research has consistently shown lower scores on social communication assessments in the first year of life, there is limited research at 12-months exploring associations between different dimensions of social communication and later language. Understanding associations between early social communication skills and language would enhance our ability to choose high priority intervention goals that will impact downstream language skills. The current study used a standardized assessment to profile social communication skills across 516 infants with a high (HL) or low likelihood (LL-Neg) for autism (84% White, 60% Male), based on the presence of a sibling with autism in the family. The primary aim of the study was to profile social communication skill development in the second year of life and to evaluate associations between social communication skills and later language. HL infants who met criteria for autism (HL-ASD, *N* = 81) demonstrated widespread reductions in social communication skills at 12-months compared to HL infants who did not meet criteria for autism (HL-Neg, *N* = 277) and LL-Neg (*N* = 158) infants. Across all infants in the study, those with better social communication skills at 12-months had better language at 24-months. However, within group analyses indicated that infants who met criteria for autism did not show this developmental coupling until 24-months-of-age at which point social communication was positively associated with downstream language skills. The cascading pattern of reduced social communication skills as well as overall significant positive associations with later language provide further evidence for the need to support developing social communication skills prior to formal autism diagnosis, a goal that could possibly be reached through pre-emptive interventions.

## Introduction

Autism spectrum disorder (ASD) is a neurodevelopmental condition, characterized by restricted, repetitive patterns of behavior and challenges in social communication (DSM-5; American Psychiatric Association, 2013). By 9- to 12-months of age, infants, who are later diagnosed autistic score lower on social communication assessments (Zwaigenbaum et al., 2005; Landa et al., 2007; Ozonoff et al., 2010; Bradshaw et al., 2020). During the early pre-diagnostic period, infants later diagnosed with autism remain consistently low, or make fewer gains on social communication skills, when compared to infants who do not meet criteria for autism (Bradshaw et al., 2021). For most children, these early observable behavioral differences in social communication consolidate into a diagnosable behavioral phenotype of autism around 24- to 36-months-of-age (Piven et al., 2017; Grzadzinski et al., 2021).

Early social communication skills are a harbinger for later language development (Wetherby et al., 2007; Delehanty et al., 2018). The cascading pattern of social communication challenges in the first two years of life experienced by infants who meet criteria for autism highlights the need to strengthen these skills (Bradshaw et al., 2021). However, current interventions for autism are typically provided following formal diagnoses. While these interventions have demonstrated some promise with improving social communication skills in autistic toddlers (Sandbank et al., 2020), there is a growing need to provide interventions that focus on early developmental trajectories rather than as a reaction to an autism diagnosis (Green et al., 2022).

### Pre-emptive interventions

Interventions provided before a diagnosis are referred to as pre-emptive interventions. Pre-emptive interventions have been reported to be feasible and acceptable by families (Green et al., 2013). Recent evidence suggests that parents can effectively implement strategies taught through pre-emptive interventions, and parent fidelity in turn leads to better child social communication outcomes (Hampton and Rodriguez, 2021; Yoder et al., 2021). Pre-symptomatic interventions have been provided for infants who have a higher familial likelihood for autism (i.e., infants who have an older autistic sibling) or for those who show early symptoms of autism (Green, 2020). These interventions have focused on social communication skills such as social play, joint attention, symbolic play, and infant vocalizations (Hampton and Rodriguez, 2021). Research has also demonstrated that parent-mediated social communication interventions have significant overall positive effects on autism symptoms during the pre-diagnostic period (Green et al., 2017), as well as autism symptom severity following a formal diagnosis (Whitehouse et al., 2021). Together, this body of research suggests that working on early social communication skills during the pre-diagnostic period might shift developmental trajectories for autistic infants.

While preemptive interventions are effective in teaching parents strategies to improve social communication outcomes, it is important to consider the impact of these interventions on language development trajectories. Fostering better language skills has been identified as a priority for interventions by the autism community (Kapp, 2020; Green et al., 2022). A few studies have explored long-term language outcomes following pre-emptive interventions, and results have been mixed (Green et al., 2017; Whitehouse et al., 2021; Yoder et al., 2021). However, proximal significant effects on parent fidelity have been found to mediate language outcomes (Watson et al., 2017; Yoder et al., 2021). More recently, a pre-emptive intervention program focused on early social communication skills resulted in significant improvements in parent-reported measures of vocabulary (Whitehouse et al., 2021). In summary, social communication skills are often targeted in pre-emptive intervention programs as they have been found to relate to better downstream language skills (Yoder et al., 2015; Delehanty et al., 2018). However, results from preemptive intervention studies suggest that long-term effects on language are mixed.

### Associations between social communication skills and downstream language in autistic toddlers

Autistic toddlers with better social communication skills measured in the second year of life also have better later language skills (Toth et al., 2006; Wetherby et al., 2007; Yoder et al., 2015; Delehanty et al., 2018). More specifically, studies have reported significant positive associations between social communication skills such as joint attention and goal-directed (i.e., intentional communication) communicative acts and later receptive and expressive language (Toth et al., 2006; Yoder et al., 2015; Delehanty et al., 2018). Intentional communication, and frequency of intentional communicative acts were positively associated with receptive and expressive language abilities (Delehanty et al., 2018). Superior communication skills related to speech (i.e., number of consonants and words used in communicative acts) in the second year of life were found to be associated with better expressive language abilities (but not receptive language abilities) at 3 years of age (Delehanty et al., 2018). However, non-verbal communication abilities (i.e., use of gestures) was found to be positively associated with receptive *and* expressive language abilities (Delehanty et al., 2018). Further, better symbolic play (i.e., using an object to represent something else such as using a block to represent a phone) and receptive vocabulary skills between 18- and 24-months-of-age were associated with lower receptive and expressive language scores a year later (Delehanty et al., 2018).

While research has reported significant positive associations between social communication skills measured in the first year of life and later language, most of the existing studies focused on a limited number of specific skills, such as joint attention (Bottema-Beutel, 2016) and use of gestures (Choi et al., 2020). Much of the research that has explored associations to later language across multiple dimensions of social communication have primarily focused on associations in the second year of life (Toth et al., 2006; Wetherby et al., 2007; Yoder et al., 2015; Delehanty et al., 2018). The current study aims to measure associations between seven specific categories of social communication skills, and later language. A fine-grained analysis will inform the selection of intervention targets that will have the most impact on later language. In addition, the current study aims to explore associations to language skills measured at 24-and-36-months-of-age. Previous research has been based on language outcomes measured at a single time point (Wetherby et al., 2007; Yoder et al., 2015; Delehanty et al., 2018). The additional language data at will enable us to measure changes in social communication and language associations over time. Overall, the present study aims to examine the timing and nature of associations between specific features of social communication and language in infants who go on to receive and autism diagnosis.

The present study is part of two multisite Infant Brain Imaging (IBIS) Network studies that prospectively followed three groups of infants: (a) typically developing infants with a low likelihood for developing autism (LL-Neg), (b) infants who have a family-history of autism but do not develop autism themselves (HL-Neg), and (c) infants who have a family-history of autism and who go on to have autism (HL-ASD). This study design provides the opportunity to prospectively explore social communication skills and their association to later language. The purposes of this study were: (a) to explore developmental trajectories of social communication skills across the three groups of infants, (b) to identify differences in social communication skills at 12-months and 24-months across the three groups of infants, (c) to explore temporal relationships between social communication skills measured at 12-and-24-months-of-age and later language, measured at 24-and-36-months-of-age and (d) to understand how social communication skills predict autism and language diagnostic outcomes.

## Methods

### Participants

This study included 516 infants form two IBIS studies. Data for the IBIS 1 study were collected between 2007 and 2012; and data for the IBIS 2 study were collected between 2012 and 2018. Data were collected at four sites: University of North Carolina at Chapel Hill; University of Washington; The Children’s Hospital of Philadelphia; and Washington University in St. Louis Data for the current study were collected between January 10th, 2008, and February 19th, 2018. Procedures for this study were approved by local Institutional Review Boards. Written informed consent was obtained from parents prior to participation.

All participants were screened, and exclusions were made for the following reasons: (1) genetic conditions or syndromes, (2) medical/neurological conditions affecting growth, development, or cognition (e.g., seizure disorder) or significant sensory impairments (e.g., vision or hearing loss), (3) birth weight <2000 g and/or gestational age <36 weeks or significant perinatal adversity and/or exposure in utero to neurotoxins, (4) contraindication for MRI, (5) predominant home language other than English, (6) adopted children or half siblings, (7) first-degree relative with psychosis, schizophrenia, or bipolar disorder (Family Interview for Genetic Studies (FIGS; Maxwell, 1992), and (8) twins.

For the IBIS 1 study, data were collected when infants were 6, 12, and 24-months-of-age. A detailed description of the IBIS 1 data collection protocol can be found in Estes et al. (2015). IBSI 2 had a variable visit schedule, where infants were seen at four of the following time points: 3, 6, 9, 12, 15, and 24. Infants from these two studies were included in the current study if they contributed at least one social communication data point and had diagnostic data at 24-months-of-age. We used 24-month diagnostic classification because a 36-month time point was not conducted across the full sample. Previous research has suggested strong diagnostic stability for autism from 24 to 36 months of age (Lord, 1995; Lord et al., 2006; e.g., Chawarska et al., 2009; Corsello et al., 2013; Guthrie et al., 2013; Shen et al., 2013; Barbaro and Dissanayake, 2017).

Infants were classified as autistic at 24-months if they met DSM-IV-TR (Diagnostic and Statistical Manual of Mental Disorders, edition IV, Text Revision; American Psychiatric Association, 2000) criteria for autistic disorder or PDD-NOS. A clinical best-estimate diagnosis of autism was made using the DSM-IV-TR criteria by expert clinicians. Clinicians used all available developmental, clinical, and parent reported measures available at 24-months to determine the diagnostic classification for each participant. These measures included the Autism Diagnostic Observation Scale (ADOS; Lord et al., 2000), The Mullen Scales of Early Learning (MSEL; Mullen, 1995), and the Vineland Adaptive Behavior Scales, Second Edition (Vineland; Sparrow et al., 2005).

Infant participants were assigned to three groups based on familial history status and diagnostic outcome. Infants who had a high likelihood for autism, by virtue of having an older sibling with autism, and met criteria for autism were assigned to the HL-ASD group (*N* = 81). Infants who had a high likelihood for autism and did not meet criteria for autism were assigned to the HL-Neg group (*N* = 277). Infants with a low likelihood for autism, who did not meet criteria for autism were assigned to the LL-Neg group (*N* = 158).

### Procedures and measures

#### Communication and symbolic behavior scales, developmental profile

The behavior sample of the CSBS (Wetherby and Prizant, 2002) was administered at 12, 15, and 24-months-of-age to assess early social communication skills. This standardized assessment uses attractive manipulatives to enable direct observations of natural play. The CSBS uses the following strategies to elicit social communication skills: communicative temptations, book sharing, pretend play, and constructive play. Administrations were videotaped and coded based on the CSBS manual. CSBS weighted raw scores for each cluster were extracted for the analyses. Raw scores were used to avoid floor effects in standard scores. The cluster scores included: emotion and eye gaze, communicative acts, use of gestures, use of sounds, use of words, understanding, and object use. Table 1 describes skills measured under each cluster of the CSBS.

All coders were trained based on guidelines described in the CSBS manual. Coders first reviewed coded practice videos with a trained coder. Next, they coded practice videos independently until they achieved 80% reliability with gold standard scoring. The gold-standard coding video was originally coded by a clinician with expertise in CSBS coding. Approximately 5% of the videos (*N* = 25) were double coded, and the coders had 86 % agreement on average (*SD* = 3.83).

Through the coding process, administration errors were identified in the symbolic and social composites, which impacted the following clusters: emotion and eye gaze, communication, gestures, understanding, and object use. Videos with administration errors were excluded from analyses for the clusters with incorrectly administered composite(s), resulting in different data sets for each CSBS score (see Tables S1 and S2 in Supplementary methods for additional information on administration errors).

#### Developmental and language measures

The MSEL (Mullen, 1995) is a standardized, direct assessment of cognitive functioning. It was administered at 12 and 24 months-of-age. A subset of infants participated in the MSEL when they were 36 months old. Subscale raw scores for receptive language (RL) and expressive language (EL) were extracted at the 24- and 36-month timepoints. Raw scores were used to avoid floor effects in standardized scores. Mullen *T*-scores were used as follows to generate two groups within the HL sample based on if the infants showed signs of early language delay (HL-Language Delay vs. HL-No Delay). These groups were made irrespective of ASD diagnostic status. Infants were determined to have signs of early language delay if their RL or EL *T-scores* fell 1.5 standard deviations below the mean (i.e. *T*-*scores* <= 35) (Swanson et al., 2017; Marrus et al., 2018). The Non-Verbal Developmental Quotient (NVDQ) was computed by averaging the age equivalent scores from the fine motor and visual reception subscales at 24-months to measure nonverbal cognitive skills. The Early Learning Composite (MSELELC) score was not used in analyses but is reported to provide an overall description of developmental functioning across the sample at 12, 24, and 36-months-of-age.

A second assessment, the Vineland (Sparrow et al., 2005) was used to measure receptive and expressive language. The Vineland, a standardized measure of adaptive functioning, was used to provide a parent-reported measure of language abilities. It was administered at 12- and 4-months-of-age *via* parent interviews. The Vineland evaluates adaptive functioning across the following domains: communication, daily living skills, socialization, and motor. Overall adaptive behavior was evaluated at 12- and 24-months of age using the Adaptive Behavior Composite (ABC). The ABC was not included in the analyses, but is reported to provide a description of adaptive functioning across the groups. EL and RL raw scores were derived at 24-months from the communication domain. While the receptive and expressive language subtests of the Vineland and MSEL measure the same construct, data from both the assessments were used in order to provide a comprehensive picture of the infant’s language abilities across different settings (lab setting vs. home environment).

### Statistical analysis plan

Table 1 includes the number of participants by group at each time point. All analyses were performed using R, version 4.1. Group differences in the trajectory of CSBS scores from 12- to 24-months-of-age were examined using the nlme package (Pinheiro et al., 2021). The mixed linear model was used to analyze the effects of group and age (in months) for all CSBS scores. The interaction effect of group by age was examined with the HL-ASD group dummy coded to be the reference group.

Next, cross-sectional analyses were completed at 12- and 24-months using the general linear model. Cross-sectional analyses were not completed at 15-months due to small sample sizes (Table 1). The main effect of group was evaluated at 12- and 24-months. Estimated marginal means were computed for follow-up group comparisons using the emmeans package (Lenth, 2016). Tukey adjustments were applied for post-hoc group comparisons.

General linear models were used to explore the effects of 12-month CSBS scores on 24-month language scores (MSEL receptive and expressive language raw scores; and Vineland receptive and expressive language raw scores); and 24-month CSBS scores on 36-month language scores (MSEL receptive and expressive language raw scores). NVDQ at 24 months was included as a covariate to explore the effects of social communication on later language while controlling for nonverbal cognitive skills. A decision was made *a priori* to explore associations between CSBS scores and later language scores in each group using the general linear model. The lm package (Pinheiro et al., 2021) in R was used for analyses using the general linear model.

Logistic regressions models were used to analyze the relationship between 12-month CSBS scores and 24-month diagnostic (HL-ASD vs. HL-Neg) and language outcomes (HL-Language Delay vs. HL-No Delay); as well as 24-month CSBS scores and 36-month language outcomes across HL infants. The LL-Neg group was not included for the language outcomes analysis due to the low occurrence of language delays in this group. Of the 141 LL-Neg infants who had language outcome data at 24-months, 9 met criteria for language delays.

For all the analyses, data collection site, maternal education, and sex of the infant were included as control variables. These control variables were selected *a priori* to account for differences in data collected across sites, associations between maternal education and child language skills (Hart and Risley, 1995), and sex differences in language acquisition (Eriksson et al., 2012). A false discovery rate (FDR) procedure was used to correct for multiple comparisons where multiple tests were done using the same outcome variable. Benjamini and Hochberg (1995) one-step model was used, and adjusted *p*-values are presented as *q*-values for significant associations.

## Results

### Sample characteristics

Table 2 contains demographic information and developmental characteristics for the HL-ASD, HL-Neg, and LL-Neg groups. Most of the participants were Caucasian (85%). The percentage of male participants was 60% for the entire sample, as compared to 78% for the HL-ASD group. Groups did not significantly differ on chronological age at the 12, 15, and 24-month time points. There was a significant positive association between group and age at 36-months (*F*(2, 240) = 10.88, *p* <0.01), such that the LL-Neg group fell significantly below the other two groups. For all analyses involving 36-month language scores, candidate age at 36-months was included as a control variable. Maternal education was significantly positively associated with all receptive and expressive language measures at 24-months (*p* < 0.05, *f*^2^ = 0.02–0.06) and included as a control variable in all models.

### Social communication development across groups

**FIGURE 1:**
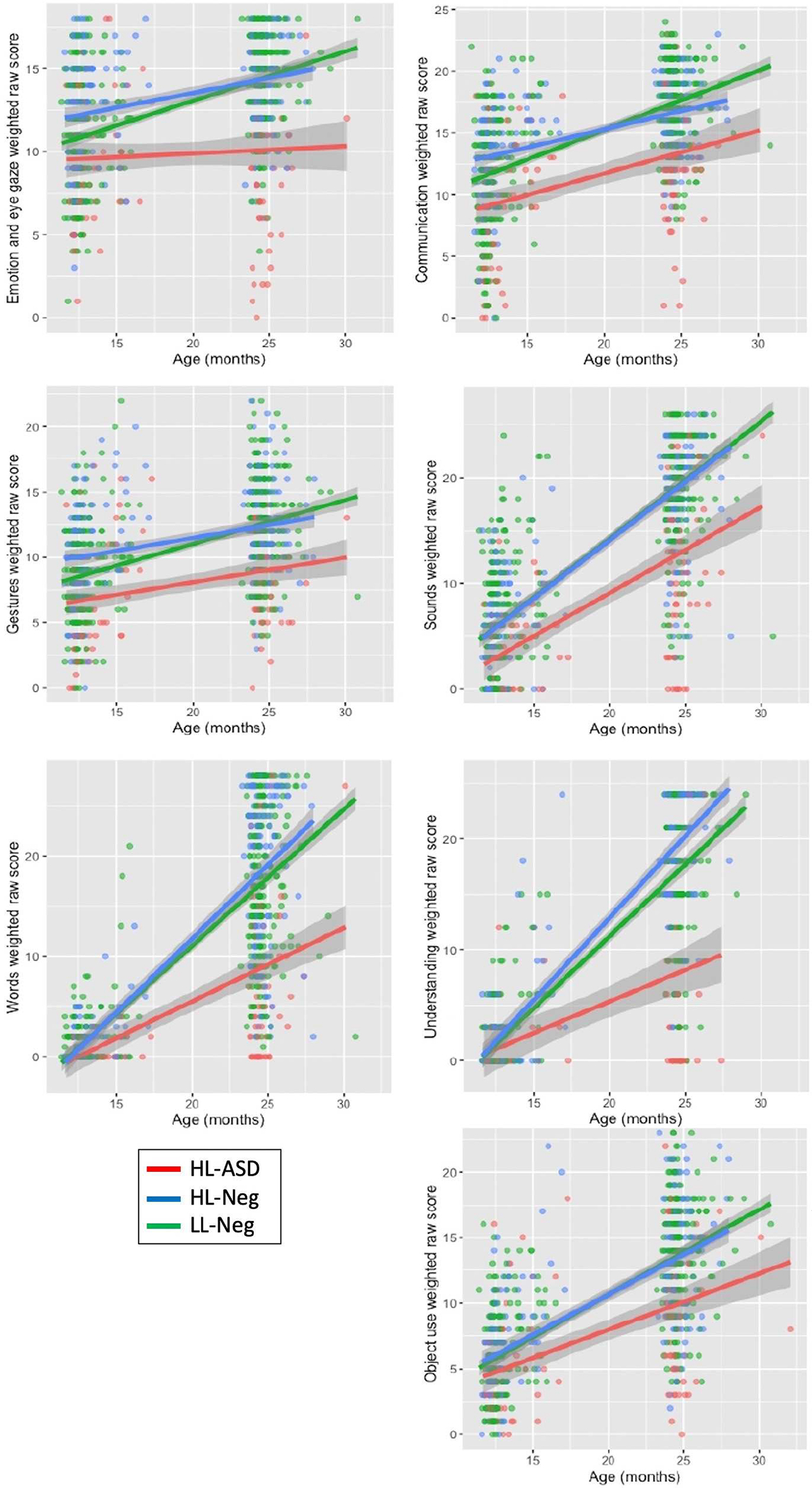
Developmental trajectory of CSBS scores by group

Mixed linear models revealed significant group by age interaction effects (*q* < *0.0*1, Table 3) for all CSBS scores. This interaction effect is represented by the widening gap over time between the HL-ASD group and the HL-Neg and LL-Neg groups (Figure 1).

#### Cross-sectional group differences at 12-months and 24-months

At 12-months, the main group effect was significant for all CSBS scores (*q* < 0.01, Table 4), except understanding and words. The HL-ASD group scored significantly below the LL-Neg group and HL-Neg groups on emotion and eye gaze, communication, gestures, and sounds (*p* < 0.01; Supplementary Figure 1, Supplementary results). The HL-ASD group scored significantly below the LL-Neg group on object use (*p* < 0.05; Supplementary Figure 1, Supplementary results). The HL-ASD group did not significantly differ from the LL-Neg group on words, understanding, and object use.

The HL-Neg group scored significantly below the LL-Neg group on emotion and eye gaze, and gestures at 12-months (*p* < 0.05; Supplementary Figure 1, Supplementary results). The HL-Neg infants did not differ significantly from the LL-Neg infants on communication, sounds, and object use.

At 24-months, for all CSBS scores, the main effect of group was significant (*q* < 0.01, Table 4). HL-ASD infants scored significantly below the other two groups on all CSBS scores (*p* < 0.01; Supplementary Figure 2, Supplementary results). The HL-Neg infants scored significantly below the LL-Neg group on understanding at 24-months. The HL-Neg and LL-Neg groups did not differ significantly from each other on any of the CSBS scores.

### Association between 12-month social communication 24-month language skills

The interaction effects of CSBS scores by group were not significantly associated with expressive language (Supplementary Table 5, Supplementary results), and were removed from subsequent models. Once the interaction term was removed, all CSBS scores were significantly positively associated with MSEL EL measures and Vineland EL measures, with effect sizes ranging from small to medium (*q* < 0.05, Table 5). All CSBS scores except for object use and emotion and eye gaze remained significantly positively associated with MSEL EL measures (*q* < 0.05, *f*^2^ = 0.05–0.17) after controlling for NVDQ. Similarly, all CSBS scores remained significantly positively associated with Vineland EL scores after controlling for NVDQ (*q* < 0.05, *f*^2^= 0.06–0.14).

The interaction effects of CSBS scores by group were not significantly associated with receptive language, except for words and Vineland RL [(*F*(2, 370) = 4.65, *p* < 0.05, *f*^2^0 = 0.03), Supplementary Table 5, Supplementary results]. Follow-up analyses indicated that in the HL-ASD & LL-Neg groups, words and Vineland RL were not significantly associated with each other. However, in the HL-Neg group there was a significant positive association between words and Vineland RL (*β*= 0.37, *t* (208) = 3.13, *p* = 0.01).

For the remaining models with non-significant interaction effects of CSBS scores by group on receptive language, the interaction term was removed from subsequent models. Once the interaction effect was removed, CSBS scores that were significantly positively associated with 24-month MSEL RL, included emotion and eye gaze and gestures (*q* < 0.05, *f*^2^= 0.02–0.11). CSBS scores that were significantly positively associated with 24-month Vineland RL included emotion and eye gaze and words (*q* < 0.01, Table 5), with small effect sizes. However, after controlling for NVDQ, none of the 12-month CSBS scores were significantly associated with MSEL RL scores. Emotion and eye gaze and words scores were significantly positively associated with Vineland RL after controlling for NVDQ (*q* < 0.01, *f*^2^= 0.06–0.12).

We also explored associations between social communication skills and later language in each group of participants. In the HL-ASD group, none of the 12-month CSBS scores were associated with MSEL and Vineland language scores (Supplementary Table 3, Supplementary results). In contrast, the HL-Neg group demonstrated significant positive associations between all CSBS scores and MSEL EL except for object use (*q* < 0.05, *f*^2^0 = 0.04–0.22). The association of words, sounds, understanding, and gestures remained significant after controlling for NVDQ. The HL-Neg group also demonstrated significant positive associations between Vineland EL and the following CSBS scores: emotion and eye gaze, communication, gestures, sounds, words, object use and Vineland EL (*q* < 0.05, *f*^2^ = 0.02–0.12). The association between Vineland EL and emotion and eye gaze, gestures, and object use and did not remain significant after controlling for NVDQ. The LL-Neg group demonstrated significant positive associations between communication, gestures, sounds, words, understanding, object use and MSEL EL (*q* < 0.05, *f*^2^0 = 0.04–0.22), which remained significant after controlling for NVDQ. The LL-Neg group also demonstrated significant positive associations between understanding and Vineland EL, and this association remained significant after controlling for NVDQ.

For MSEL-RL, the HL-Neg group demonstrated significant positive associations with emotion eye gaze, and gestures (*q* < 0.05, *f*^2^ =0.04–0.06). Vineland RL in the HL-Neg group was significantly positively associated with words and communication (*q* < 0.05, *f*^2^ = 0.02–0.08). However, none of these associations in the HL-Neg group remained significant after controlling for NVDQ, except for words and Vineland RL. The LL-Neg group did not demonstrate any significant associations between 12-month CSBS scores and 24-month Mullen and Vineland RL scores.

### Association between 24-month social communication 36-month language skills

The CSBS scores by group interaction effects were significant for MSEL EL and emotion and eye gaze (*q* <0.05; Supplementary Table 6, Supplementary results). Follow-up groupwise analyses indicated that the HL-ASD group demonstrated a significant positive association (*β*= 0.81, *t*(36) = 3.51, *p* <0.01, *f*^2^ = 0.42), whereas the associations were not significant for the HL-Neg group (*β*=−0.07, *t*(102) =−0.40, *q* = 0.69) and LL-Neg groups (*β*= −0.22, *t*(36) = −0.75, *q* = 0.46). The interaction effects of CSBS scores by group on MSEL EL were not significant (Supplementary Table 6, Supplementary results) for all other models and were removed from subsequent models. Once the interaction term was removed, emotion and eye gaze, communication, gestures, sounds, words, and understanding were significantly positively associated with MSEL EL measures, with small to large effect sizes (*q* < 0.05, Table 6). All positive associations remained significant after controlling for NVDQ (*q* < 0.01, *f*^2^ = 0.08–0.69, Table 6).

For MSEL RL, the CSBS scores by group interaction effects were significant for emotion and eye gaze and understanding (*q* < 0.05; Supplementary Table 6, Supplementary results). Follow-up groupwise analyses indicated that the HL-ASD group demonstrated a significant positive associations between emotion and eye gaze and MSEL RL (*β*= 0.61, *t* (38) = 2.76, *p* <0.01, *f*^2^= 0.30), whereas the associations were not significant for the HL-Neg group (*β* =−0.10, *t* (102) =−0.54, *q* = 0.59) and LL-Neg groups (*β* =−0.20, *t* (36) =−0.61, *q* = 0.54). Associations between understanding and MSEL RL was significant for HL-ASD infants (*β*= 0.59, *t* (24) = 4.07, *q* <0.01, *f*^2^_=_ 0.61) and HL-Neg infants (*β*= 0.20, *t* (69) = 3, *q* <0.01, *f*^2^_=_ 0.14), but not for LL-Neg infants (*β*= 0.53, *t* (31) = 2.75, *q* < 0.07).

The interaction effects of remaining CSBS scores (communication, gesture, sounds, words, understanding, object use) by group on MSEL RL were not significant (Supplementary Table 6, Supplementary results) for all other models and were removed from subsequent models. Once the interaction term was excluded, CSBS scores that were significantly associated with 36-month MSEL RL included emotion and eye gaze, communication, sounds, words, and understanding (*q* < 0.01, Table 6), with effect sizes ranging from small to large. These associations remained significant after controlling for NVDQ (*q* < 0.01, Table 6). Gestures scores at 24-months were also significantly positively associated with MSEL RL at 36-months (*F*(1, 379) = 4.86, *q* < 0.03, *f*^2^= 0.06), however this association did not remain significant after controlling for NVDQ.

Follow-up groupwise analyses revealed unique patterns for 24-month social communication and 36-month language associations. The HL-ASD group demonstrated significant associations between emotion-eye gaze, sounds, words, understanding and MSEL-EL (*q* < 0.05, *f*^2^ = 0.27–0.41), with emotion-eye gaze, sound, and word associations continuing to remain significant after controlling for NVDQ (Supplementary Table 6, Supplementary results). HL-Neg group demonstrated significant associations between sounds, words, understanding and MSEL EL (*q* <0.05, *f*^2^ = 0.09–0.25). MSEL EL and sounds, words, and communication were significantly positively associated after controlling for NVDQ. For MSEL-RL, HL-ASD demonstrated significant associations for emotion-eye gaze, sounds, words, understanding (*q* < 0.05, *f*^2^ =0.13–0.56), however, none of these associations remained significant after controlling for NVDQ. HL-Neg group demonstrated significant associations for sounds, words, understanding and MSEL-RL (*q* < 0.01, *f*^2^ = 0.13–0.15). These associations in the HL-Neg group continued to remain significant after controlling for NVDQ. In contrast, LL-Neg group did not demonstrate significant associations to 36-month MSEL language scores.

### Social communication skills as predictors of autism diagnoses and language delays

Logistic regression was used to determine if CSBS scores predicted later autism diagnoses and language delay status. These analyses were conducted within the HL infants only. Of the HL infants 22% met criteria for signs of early language delay at 24 and 36 months (Table 7).

The models exploring 12-month CSBS scores as predictors of 24-month autism diagnosis and language delay in the high-likelihood infants did not reveal any significant associations (Table 8). However, the models exploring 24-month CSBS scores as predictors of 36-month language delay revealed a significant negative association between 24-month understanding scores and 36-month language delay outcome (*q* < 0.01, Table 9). The estimated odds ratio indicated that, holding all other social communication scores constant, the odds of a language delay increased by 0.85 times (95% CI [0.78, 0.91]) per one unit decrease in understanding scores. The sensitivity of the model was 45%, which meant that the model correctly classified infants who went on to have a language delay 45% of the time using understanding scores. The model had a specificity of 95%, which meant infants who did not go on to have a language delay were correctly classified 95% of the time using understanding scores. None of the other 24-month social communication scores predicted autism diagnosis or language delays.

## Discussion

This prospective study explored social communication skills and their associations to language in infants later diagnosed autistic. Social communication skills were evaluated at 12, 15, and 24-months in a large sample of infants that were either: (a) typically developing infants with no family history of autism (LL-Neg), (b) infants with a family history of autism who were later diagnosed autistic (HL-ASD), or (c) infants with a family history of autism who were not later diagnosed autistic (HL-Neg). The clinical implications for early identification and intervention for autism are discussed below.

HL-ASD infants demonstrated lower scores on social communication assessments at 12-months-of-age across widespread domains, and these early differences became more pronounced in the second year of life. These findings add to existing research reporting that social communication difficulties are detectable using standardized assessments as early as 9- to 12-months-of-age (Bradshaw et al., 2021). HL-ASD infants demonstrated unique social communication developmental trajectories in the second year of life. As a group, they did not make significant gains on the emotion and eye gaze cluster. On the gesture cluster, HL-ASD made parallel gains when compared to the LL-Neg infants in the second year of life and remained significantly below LL-Neg and HL-Neg infants throughout. Overall, HL-ASD infants remained consistently low on emotion and eye gaze and gestures, a finding that corroborates previous reports in infants between 9- and 12-months-of-age (Rozga et al., 2011; Iverson et al., 2018; Stallworthy et al., 2021). In contrast, HL-ASD infants demonstrated a growing gap on the sounds and object use clusters. They scored significantly below the other two groups at 12-months-of-age, but this gap widened over time, with the HL-ASD group making fewer gains when compared to the other two groups. Group level differences in the areas of words and understanding were late to emerge. While HL-ASD infants did not score significantly below the other two groups at 12-months-of-age, they scored significantly below at 24-months-of-age. Similar patterns of social communication development (i.e., consistently low, growing gap, and late emerging) were reported by Bradshaw et al. (2021) between 9- and 12-months-of-age. Overall, these developmental trajectories suggest that social communication skills are an ideal target for preemptive interventions (i.e., interventions that are provided prior to formal autism diagnosis) as the goal through these interventions is to reshape symptom trajectories in the prodromal period.

Across all infants, better scores on all CSBS clusters at 12-months-of-age were related to better expressive language scores at 24-months of age. Better emotion and eye gaze and gesture scores were associated with better 24-month receptive language scores. Further, better scores on the sounds, words, and understanding clusters at 24-months-of-age were associated with better 36-month receptive and expressive language scores. In addition, infants who had better emotion and eye gaze and communication skills at 24-months also had higher 36-month expressive language scores.

This current study is the first to explore associations between a wide range social communication skills and language in infants with a high likelihood for autism as early as 12-months-of-age. Extending this research to younger ages has revealed shifts in associations between early social communication and later language, unique to infants later diagnosed autistic.

In the HL-ASD group, none of the CSBS scores at 12-months were associated with language at 24-months-of-age. In contrast, CSBS scores as early as 12-months-of age were significantly positively associated with 24-month language in the HL-Neg and LL-Neg groups. This functional association did not emerge in the HL-ASD group until 24-months-ofage, at which point emotion-eye gaze, sounds, words, and understanding were significantly positively associated with 36-month receptive and expressive language. This finding is consistent with previous research that has reported that social communication skills measured 20-months-of-age and beyond are associated with downstream language skills in autistic toddlers (Yoder et al., 2015; Delehanty et al., 2018). It is likely that HL-ASD infants did not demonstrate this association at 12-months-of-age because they had not yet acquired the relevant skills that are developmentally “upstream” from language. Once these children made advancements in the areas of emotion and eye gaze, sounds, words, and understanding, associations to later language emerged. An additional possibility is that at 12-months, skills other than social communication support later language development in the HL-ASD group. While the current study focused on social communication skills, from a “developmental cascades” perspective, it is possible that development in other domains (such as motor skills, temperament, sleep, visual attention) may impact downstream language abilities (Bradshaw et al., 2022). Future research should explore these cascading effects on language in order to elucidate alternative pathways to language in HL-ASD infants.

Pecukonis et al. (2022) recently reported surprising group differences in the association between caregiver-reported gestures at 12-months and MSEL language skills at 36 months. Significant positive associations were reported between 12-month gesture use and language in HL-Neg infants, which corroborated findings from the current study. However, they also reported negative associations between 12-month gesture and language in HL-ASD infants. The current study did not find significant negative associations between gesture and language in HL-ASD infants. These differences could be attributed to differences in measures used for gestures (parent-report vs. direct assessment) or differences in the timepoint for language skills (36 vs. 24 months). Future efforts should aim to replicate findings from the current study and Pecukonis et al. (2022) to provide clarity on the developmental relationship between gestures and language for infants who develop autism.

Although 12-month social communication skills were not associated with later language in the HL-ASD group, 24-month social communication skills were associated with later language, and this suggests that supporting early social communication development may relate to better downstream language skills. Further, HL-Neg infants are more likely to demonstrate language delays than LL-Neg infants (Miller et al., 2016; Marrus et al., 2018); and our findings revealed that supporting social communication skills in this group may relate to better language skills as well.

This study is also the first to explore the utility of social communication profiles in predicting autism diagnoses and language delays in infants with a high likelihood for autism. At 24-months, understanding scores significantly predicted 36-months language delay outcomes. While understanding scores accurately predicted high-likelihood infants who met criteria for language delays only 45% of the time; they accurately predicted infants who did not meet criteria for language delays 95% of the time. The understanding cluster measures receptive vocabulary. Our findings suggest that early receptive vocabulary can be used to screen out infants within the high likelihood group who do not need additional language interventions. Further, these findings also suggest that providing interventions in response to early receptive vocabulary developmental trajectories needs to be a key focus of preemptive interventions.

Overall, these findings suggest that preemptive interventions should follow a personalized, developmental approach and capitalize on existing social communication skills to increase bouts of shared engagement, use of intentional communication, and gesture use (i.e., skills measured in the social composite). During instances of intentional communication, the use of sounds and words can be encouraged using parental scaffolding. While infants later diagnosed autistic did not demonstrate significant differences at the group level on symbolic skills (i.e., understanding and object use) at 12-months-of-age, it is important to monitor these skills and provide interventions tailored to support symbolic skills as challenges in this area may emerge later in development.

While preemptive interventions for autism are yet to demonstrate conclusive evidence of efficacy, preliminary research reports are promising (Hampton and Rodriguez, 2021). Preemptive interventions have been successfully implemented for infants recruited based on a higher familial likelihood for autism (Green et al., 2015, 2017; Yoder et al., 2021) as well as community-based screening approaches (Watson et al., 2017; Whitehouse et al., 2021). Recent research has demonstrated that parent-implemented preemptive interventions have significant effects on child autism symptom severity and parent-reported receptive and expressive vocabulary (Whitehouse et al., 2021). Although further research is required to gather conclusive evidence of improved child outcomes, this research suggests that parents are able to implement intervention strategies with fidelity and this in turn may relate to better social communication outcomes (Watson et al., 2017; Hampton and Rodriguez, 2021; Yoder et al., 2021). Our findings provide evidence that improving social communication outcomes could confer better downstream language skills. However, in order to establish causal relationships, preemptive intervention research should explore downstream developmental effects of proximal social communication outcomes (Chawarska et al., 2009)

A limitation of this study is that the sample did not include infants with other developmental delays. Hence, the profile of social communication skills reported in this study may not be specific to autism. It should be noted, however, that approximately 30% of high likelihood infants who do not meet criteria for autism demonstrate other clinical concerns at school age, such as broader autism phenotype (BAP), Attention-Deficit/Hyperactivity Disorder (ADHD), and speech and language problems (Miller et al., 2016). Including a comparison group of infants with other developmental disabilities (such as Down’s syndrome) may reveal developmental trajectories and language associations that are unique to autism. This study also had a limited sample size at 15-months. Future research should include multiple sampling points across developments to accurately characterize social communication developmental trajectories. Another important future research goal is extended follow-up of these infants. This will elucidate long-term (i.e., school-age and beyond) effects of early social communication delays. It is also important to consider heterogeneity in the early development of autism, which could impact diagnostic classification over time. While 24-month diagnostic classification is stable (Chawarska et al., 2009; Corsello et al., 2013; Guthrie et al., 2013; Barbaro and Dissanayake, 2017), extended follow-up can account for changes in diagnostic classification across groups. An additional limitation of this study is the lack of racial diversity in the sample. Future research should aim to recruit a racially diverse sample as research has reported racial disparities in the timing of autism diagnoses and access to interventions, particularly in African American autistic children (Constantino et al., 2020).

### Conclusions

Infants later diagnosed autistic demonstrate widespread challenges with social communication skills as early as 12-months-of-age and this gap in social communication skills was more pronounced in the second year of life. Better social communication skills at 12-months-of-age were not associated with better with downstream receptive and expressive language skills in this group. Contrastingly, infants who did not meet criteria for autism demonstrated significant positive associations between early social communication skills and later language. This functional association only emerged in autistic infants in the second year of life, at which point 24-month social communication skills were positively associated with 36-month language skills. Further, understanding (i.e., receptive vocabulary) scores at 24-months-of-age significantly predicted language delays in infants with an older autistic sibling. Taken together, these findings support the need for preemptive interventions that are designed to respond to early developmental trajectories that consolidate into an autism diagnosis. Social communication skills, particularly sharing attention, goal-directed communication (using gestures, sounds, and words), and understanding are ideal preemptive intervention targets as they related to better downstream language abilities.

## Supplementary Material

Supplementary Methods

## Figures and Tables

**TABLE 1:** OverView of social communication skills measures in the CSBS composites.

Composite	Cluster	Skills measured

Social	Emotion and eye gaze	Shifting gaze between object and communicative partner Sharing positive affect with communicative partner Responding to joint attention (RJA)
	Communication	Frequency and variety of intentional communicative acts (e.g., requesting, refusing, seeking comfort)
	Gestures	Use of conventional gestures (e.g., pointing, nodding, showing) Use of distal gestures (i.e., gestures that do not involve touching an object or person)
Speech	Sounds	Use of sounds in communicative acts
	Words	Use of words in communicative acts
Symbolic	Understanding	Comprehension of object names, body part names, and person names (i.e., receptive vocabulary)
	Object Use	Use of objects during symbolic play Constructive play (i.e., stacking blocks)

**TABLE 2:** Descriptive data for study sample by Group.

Variable	HL-ASD (*N* = 81)	HL-Neg(*N* = 277)	LL-Neg (*N* = 158)	Test statistic

% Male	78	55	59	χ^2^ = 14.10, *p* < 0.01
**Maternal education**				χ ^2^ = 34.22, *p* < 0.01
High school diploma (%)	42	31	16	
College degree (%)	31	45	39	
Graduate degree (%)	27	24	45	
**Paternal education**				χ ^2^ = 11.76, *p* = 0.02
High school diploma (%)	43	32	23	
College degree (%)	33	38	41	
Graduate degree (%)	22	30	36	
Race				χ ^2^ = 12.91, *p* =0.23
White (%)	88	91	87	
African American (%)	1	3	6	
Asian (%)	1	1	1	
Multiracial (%)	15	9	11	
*N* at 12-months	62	223	115	
*N* at 15-months	9	15	10	
*N* at 24-months	65	241	129	
Age at 12-months	12.8 (0.73)	12.6 (0.66)	12.7 (0.83)	*F* = 1.14, *p* =0.32
Age at 15-months	15.8 (0.81)	15.4 (0.45)	15.5 (0.40)	*F* = 1.72, *p* =0.19
Age at 24-months	24.8 (1.30)	24.7 (0.91)	24.8 (0.87)	*F* = 13.56, *p* =0.87
Age at 36-months	39.7 (4.58)	39.5 (4.90)	43.8 (8.53)	*F* = 10.88, *p* <0.01
**MSEL-ELC**				
12-months	92.36 (14.97)	101.18 (2.34)	106.39 (11.48)	*F* = 31.83, *p* <0.01
24-months	80.18 (17.13)	102.04 (15.60)	110.27 (15.22)	*F* = 82.80, *p* <0.01
36-months	83.24 (21.29)	103.97 (18.03)	111.69 (15.31)	*F* = 37.13, *p* <0.01
**MSEL NVDQ**				
12-months	109.38 (13.15)	113.31 (12.68)	116.39 (11.30)	*F* = 5.43, *p* <0.01
24-months	87.91 (13.04)	101.98 (12.92)	108.75 (13.16)	*F* = 71.99, *p* <0.01
36-months	87.28 (19.83)	105.62 (16.47)	109.14 (13.21)	*F* = 29.91, *p* <0.01
**MSEL expressive language raw scores**				
12-months	11.01 (2.65)	12.27 (2.58)	13.01 (2.49)	*F* = 5.73, *p* <0.01
24-months	18.01 (5.42)	22.43 (4.17)	23.81 (4.05)	*F* = 33.45, *p* <0.01
36-months	29.24 (7.08)	33.96 (4.74)	38.23 (5.16)	*F* = 30.29, *p* <0.01
**MSEL receptive language raw scores**				
12-months	11.67 (2.39)	12.56 (2.06)	13.76 (1.86)	*F* = 12.82, *p* <0.01
24-months	18.57 (6.69)	25.27 (3.35)	26.56 (3.07)	*F* = 87.20, *p* <0.01
36-months Vineland ABC	29.30 (7.30)	33.56 (4.80)	38.14 (6.26)	*F* = 26.31, *p* <0.01
12-months	89.47 (14.39)	96.30 (13.98)	100.48 (9.54)	*F* = 20.06, *p* <0.01
24-months	88.93 (9.75)	101.51 (10.90)	103.96 (11.43)	*F* = 41.63, *p* <0.01
**vineland expressive language raw scores**				
12-months	16.14 (4.53)	19.00 (5.26)	20.40 (3.94)	*F* = 16.71, *p* <0.01
24-months	33.44 (12.76)	45.93 (12.63)	50.01 (11.54)	*F* = 14.78, *p* <0.01
**Vineland receptive language raw scores**				
12-months	9.55 (3.43)	11.31 (3.25)	12.46 (3.31)	*F* = 38.56, *p* <0.01
24-months	18.02 (6.24)	24.07 (3.65)	25.25 (3.44)	*F* = 77.16, *p* <0.01

**TABLE 3:** Longitudinal mixed linear model with group by age interaction effects.

CSBS scores	Group*age interaction			
	*df* 1	*df* 2	*F*	*q*

Emotion and eye gaze (*N* = 494)	2	321	16.26	<0.01*
Communication (*N* = 511)	2	347	8.56	<0.01*
Gestures (*N* = 511)	2	347	6.59	<0.01*
Sounds (*N* = 511)	2	347	8.23	<0.01*
Words (*N* = 511)	2	347	29.08	<0.01*
Understanding (*N* = 409)	2	159	36.85	<0.01*
Object use (*N* = 485)	2	289	6.07	0.01*

* Significant interaction that survived adaptive FDR procedure.

**TABLE 4:** *Post-hoc* cross-sectional analysis exploring the main effect for group.

CSBS scores	HL-ASD (a)		HL-Neg (b)		LL-Neg (c)		Overall group comparison					*Post-Hoc* comparisons

	EMM	SE	EMM	SE	EMM	SE	*df*	SS	*F*	*q*	*f* ^2^	

**12-months**												
Emotion eye gaze (*N* = 378)	9.63	0.44	10.95	0.23	12.06	0.33	2	213.90	9.88	<0.01*	0.07	a < b < c
Communication (*N* = 396)	9.07	0.56	11.80	0.29	12.89	0.42	2	549.60	15.17	<0.01*	0.10	a < b, c
Gestures (*N* = 396)	6.57	0.47	8.53	0.24	9.92	0.35	2	415.30	16.45	<0.01*	0.10	a < b < c
Sounds (*N* = 396)	3.52	0.59	6.34	0.31	5.73	0.44	2	375.50	9.32	< 0.01*	0.06	a < b, c
Words (*N* = 396)	0.40	0.24	1.00	0.13	0.95	0.18	2	17.86	2.62	0.25	0.02	a, b, c
Understanding (*N* = 262)	1.04	0.54	1.62	0.29	2.07	0.41	2	25.26	1.67	0.42	0.01	a, b, c
Object use (*N* = 334)	4.84	0.47	5.91	0.26	6.29	0.36	2	69.90	3.14	0.05	0.02	a, b, c
**24-months**												
Emotion and eye gaze (*N* = 410)	9.96	0.38	14.49	0.19	14.34	0.27	2	1,016.00	60.18	< 0.01*	0.30	a < b, c
Communication (*N* = 432)	13.40	0.43	17.50	0.22	16.70	0.31	2	853.30	37.03	< 0.01*	0.19	a < b, c
Gestures (*N* = 432)	9.08	0.45	12.59	0.23	12.53	0.32	2	651.90	26.63	< 0.01*	0.14	a < b, c
Sounds (*N* = 432)	13.00	0.69	19.60	0.36	19.20	0.50	2	2,246.90	37.73	< 0.01*	0.19	a < b, c
Words (*N* = 432)	9.55	0.99	17.75	0.51	18.78	0.71	2	4,008.20	33.43	< 0.01*	0.18	a < b, c
Understanding (*N* = 289)	8.27	1.03	17.44	0.54	19.70	0.66	2	3,745.00	45.39	< 0.01*	0.36	a < b < c
Objectuse (*N* = 413)	10.20	0.56	13.60	0.28	13.70	0.41	2	608.00	16.72	< 0.01*	0.10	a < b, c

* Significant interaction that survived adaptive FDR procedure.

**TABLE 5:** General liner model exploring main effects of social communication skills measured at 12-months on language abilities measured at 24-months.

CSBS scores	Main effect of CSBS scores				Main effect of CSBS scores with NVDQ			
	SS	*F*	*q*-value	*f* ^2^	SS	*F*	*q*-value	*f* ^2^

**MSEL EL**								
Emotion eye gaze (*N* = 376)	88.2	5.13	0.02*	0.06	12.3	0.86	0.35	0.07
Communication (*N* = 393)	151	8.87	<0.01*	0.10	72.5	5.06	0.03*	0.11
Gestures (*N* = 393)	172.7	10.18	<0.01*	0.11	88.6	6.20	0.02*	0.13
Sounds (*N* = 393)	312.8	18.85	<0.01*	0.14	198.2	14.17	<0.01*	0.16
Words (*N* = 393)	136.8	8.02	<0.01*	0.04	126.6	8.93	<0.01*	0.05
Understanding (*N* = 260)	311.8	19.34	<0.01*	0.10	247.8	17.93	<0.01*	0.12
Object use (*N* = 332)	190.9	11.70	<0.01*	0.06	48.3	3.44	0.07	0.07
**Vineland EL**								
Emotion eye gaze (*N* = 368)	1,624	11.52	<0.01*	0.09	745	5.72	0.02*	0.10
Communication (*N* = 384)	2,497	17.95	<0.01*	0.13	1,786	13.98	<0.01*	0.14
Gestures (*N* = 384)	1,676	11.86	<0.01*	0.11	1,101	8.49	<0.01*	0.12
Sounds (*N* = 384)	2,751	19.87	<0.01*	0.11	1,951	15.32	<0.01*	0.12
Words (*N* = 396)	1,641	11.61	<0.01*	0.06	1,581	12.32	<0.01*	0.06
Understanding (*N* = 253)	1,816	13.64	<0.01*	0.08	1,430.2	11.87	<0.01*	0.09
Object use (*N* = 325)	1,689	12.10	<0.01*	0.06	669	5.14	0.02*	0.06
**MSEL RL**								
Emotion eye gaze (*N* = 376)	112.9	7.55	0.04*	0.11	18.5	1.58	0.44	0.15
Communication (*N* = 393)	63.8	4.34	0.05	0.11	15	1.31	0.44	0.14
Gestures (*N* = 393)	91.4	6.25	0.04*	0.11	30.2	2.66	0.36	0.14
Sounds (*N* = 393)	22.5	1.52	0.25	0.06	0.60	0.05	0.82	0.08
Words (*N* = 393)	3.9	0.26	0.61	0.02	2.3	0.20	0.82	0.02
Understanding (*N* = 260)	88.9	5.31	0.05	0.06	52.3	3.85	0.35	0.07
Object use (*N* = 332)	67.5	4.41	0.05	0.05	1.5	0.11	0.82	0.06
**Vineland RL**								
Emotion eye gaze (*N* = 368)	223.6	13.85	0.01*	0.11	136.4	8.76	0.01*	0.12
Communication (*N* = 384)	55.0	3.41	0.11	0.07	28.2	1.83	0.24	0.08
Gestures (*N* = 383)	39.0	2.40	0.13	0.06	17.6	1.14	0.33	0.07
Sounds (*N* = 384)	78.1	4.85	0.06	0.06	44.4	2.89	0.21	0.06
Words (*N* = 384)	149.5	9.40	<0.01*	0.06	143.5	9.49	0.01*	0.06
Understanding (*N* = 253)	49.3	3.00	0.12	0.03	36.6	2.28	0.23	0.03
Object use (*N* = 325)	35.9	2.21	0.14	0.02	6.9	0.43	0.51	0.02

* Significant main effect that survived adaptive FDR procedure.

The degree of freedom for all models was 1.

**TABLE 6:** General liner model exploring main effects of social communication skills measured at 24-months on language abilities measured at 36-months.

CSBS scores	Main effect of CSBS scores				Main effect of CSBS scores with NVDQ			
	SS	*F*	*q*-value	*f* ^2^	SS	*F*	*q*-value	*f* ^2^

**MSEL EL**								
Emotion eye gaze (*N* = 194)	232.3	10.79	<0.01*	0.24	166.4	8.99	<0.01*	0.28
Communication (*N* = 200)	298.1	14.22	<0.01*	0.18	262.9	14.53	<0.01*	0.21
Gestures (*N* = 200)	100.6	4.57	0.04*	0.07	72.7	3.81	0.06	0.08
Sounds (*N* = 200)	935.9	53.17	<0.01*	0.56	715.54	45.57	<0.01*	0.63
Words (*N* = 200)	814.4	44.64	<0.01*	0.48	584.98	35.69	<0.01*	0.54
Understanding (*N* = 144)	486.87	23.54	<0.01*	0.63	288.36	15.19	<0.01*	0.69
Object use (*N* = 192)	72.9	3.34	0.06	0.08	33.5	1.74	0.19	0.10
**MSEL RL**								
Emotion eye gaze (*N* = 194)	205.6	9.62	<0.01*	0.22	138.4	7.83	<0.01*	0.27
Communication (*N* = 200)	169.3	7.88	<0.01*	0.13	139.7	7.89	<0.01*	0.16
Gestures (*N* = 200)	106	4.86	0.03*	0.06	73.9	4.09	0.05	0.07
Sounds (*N* = 200)	530.6	27.10	<0.01*	0.36	342.72	20.6	<0.01*	0.42
Words (*N* = 200)	531.1	27.13	<0.01*	0.31	321.9	19.23	<0.01*	0.37
Understanding (*N* = 144)	679.02	36.84	<0.01*	0.82	426.42	25.75	<0.01*	0.92
Object use (*N* = 192)	76.1	3.53	0.06	0.07	31.9	1.74	0.19	0.09

* Significant main effect that survived adaptive FDR procedure.

The degree of freedom for all models was 1.

**TABLE 7:** Number of infants identified to have language delays among HL-infants by visit.

	24-months	36-months

HL	309	192
HL-language delay	69 (22%)	42 (22%)
HL-no delay	240 (78%)	150 (78%)

**TABLE 8:** Logistic regression analysis exploring main effects of social communication skills measured at 12-months on diagnostic and language outcomes measured at 24-months in HL-infants.

CSBS scores	Main effect of CSBS scores				
	Estimate	SE	*z*-value	*q*-value	OR

**Autism diagnostic outcome (*N* = 163)**					
Emotion eye gaze	−0.02	0.07	−0.37	0.93	0.97
Communication	−0.12	0.06	−1.99	0.33	0.89
Gestures	−0.04	0.07	−0.57	0.93	0.96
Sounds	−0.02	0.06	−0.26	0.93	0.98
Words	−0.04	0.20	−0.22	0.93	0.95
Understanding	−0.01	0.08	−0.09	0.93	0.99
Object use	−0.02	0.07	−0.24	0.93	0.98
**Language delay diagnostic outcome (*N* = 139)**					
Emotion eye gaze	−0.08	0.09	−0.93	0.49	0.92
Communication	−0.09	0.07	−1.30	0.48	0.91
Gestures	−0.05	0.08	−0.56	0.67	0.95
Sounds	0.09	0.08	1.09	0.48	1.09
Words	−0.39	0.29	−1.35	0.48	0.68
Understanding	−0.15	0.13	−1.14	0.48	0.86
Object use	−0.03	0.09	−0.37	0.71	0.97

**TABLE 9:** Logistic regression analysis exploring main effects of social communication skills measured at 24-months on language outcomes measured at 36-months in HL-infants.

CSBS scores	Main effect of CSBS scores				
	Estimate	SE	*z* value	*q* value	OR

**Language delay diagnostic outcome (*N* = 103)**					
Emotion eye gaze	0.00	0.10	0.03	0.98	1.00
Communication	0.29	0.16	1.79	0.17	1.34
Gestures	−0.13	0.13	−1.00	0.45	0.88
Sounds	−0.21	0.09	−2.32	0.07	0.81
Words	−0.01	0.07	−0.21	0.97	0.99
Understanding	−0.16	0.05	−3.21	<0.01*	0.85
Object use	−0.14	0.10	−1.42	0.27	0.87

## Data Availability

The raw data supporting the conclusions of this article will be made available by the authors, without undue reservation.
