# Supplementary material for: Are early social communication skills a harbinger for language development in infants later diagnosed autistic?—A longitudinal study using a standardized social communication assessment
Source: Front Commun (Lausanne). Author manuscript; Available in PMC 2023 May 9. (PMC10167971; doi:10.3389/fcomm.2022.977724)
Supplement: Supplementary Methods [file NIHMS1877054-supplement-Supplementary_Methods.pdf]

1 Supplementary Methods

As of August 10th, 2021, data were available for 516 infants. CSBS data were collected between January 10th, 2008 and February 19th, 2018.

There were 22 sibling pairs that met inclusion criteria (i.e., there was one proband and two subjects in the study from the same family). One infant from each pair was selected to be included in the current analyses based on the following a priori criteria: (a) if only one infant contributed CSBS data at 12-months of age, they were included, (b) if both infants had valid data at 12-months of age, the infant with CSBS data at 24-months of age was included, (c) if both infants contributed data at the 12 and 24-month time points, the infant in the HL-ASD group was prioritized, and (d) if both infants contributed data at the 12 and 24-month time points, and the infants did not differ in group membership, the infant with language outcome data at 24-months was included.

For the general linear models exploring associations between 12-month social communication scores and 24-month receptive and expressive language scores, the 24-month non-verbal development quotient (NVDQ) was used to account for effects of non-verbal cognition on language. The NVDQ score was extracted from the MSEL by averaging scores from the non-verbal subscales and dividing by chronological age.

$$\text{NVDQ} = \frac{\text{Average of visual reception age-equivalent and fine motor age-equivalent}}{\text{Chronological age}} \times 100$$

Through the process of scoring CSBS videos, administration errors were identified in the symbolic and social composites. The following administration errors were noticed: insufficient RJA probes, incorrect number of receptive vocabulary probes, and incorrectly administered constructive play probe. Videos with administration errors were excluded from analyses for the total scores and incorrectly administered composite(s). Table S1 reports the number of observations (videos) included by group and visit for each composite, and Table S2 reports the number of videos with administration errors for each composite.

Table S1. *Number of observations for each visit by group*

CSBS scores	HL-ASD (*N* = 81)	HL-Neg (*N* = 277)	LL-Neg (*N* = 158)
**12- month visit**			
Social[1]	61	211	108
Speech[2]	62	223	115
Symbolic[3]	39	137	69
**15-month visit**			
Social	9	13	9
Speech	9	15	10
Symbolic	9	8	5
**24-month visit**			
Social	61	229	123
Speech	65	241	129
Symbolic	41	145	96

[1] The social composite included emotion and eye gaze, communication and gestures
[2]The speech composite included sounds and words
[3]The symbolic composite included understanding and object use

Table S2. *Number of videos excluded due to administration errors from each CSBS composite by visit*

Variable (Total number of videos = 908)	Number of videos excluded for administration errors
**12- month visit (*n* = 413)**	
CSBS Social[1]	33
CSBS Speech[2]	13
CSBS Symbolic[3]	177
**15-month visit (*n* = 35*)***	
CSBS Social	4
CSBS Speech	1
CSBS Symbolic	16
**24-month visit (*n* = 460*)***	
CSBS Social	47
CSBS Speech	25
CSBS Symbolic	183

[1] The social composite included emotion and eye gaze, communication and gestures
[2]The speech composite included sounds and words
[3]The symbolic composite included understanding and object use

Supplementary Results

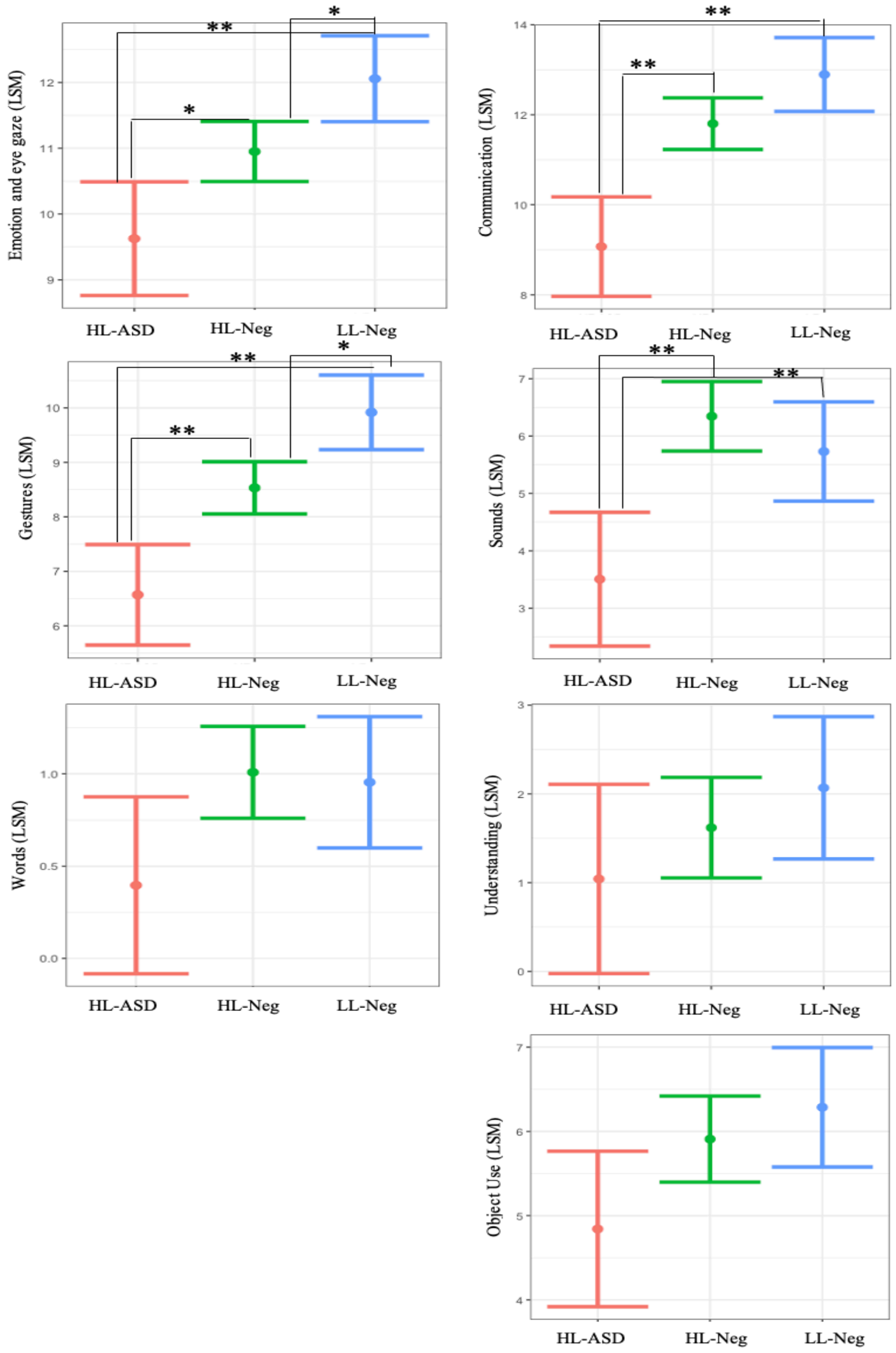

**$p < .001$; *$p < .05$

Figure S1. *Least square means (LSM) of CSBS cluster scores by group at 12-months-of-age*

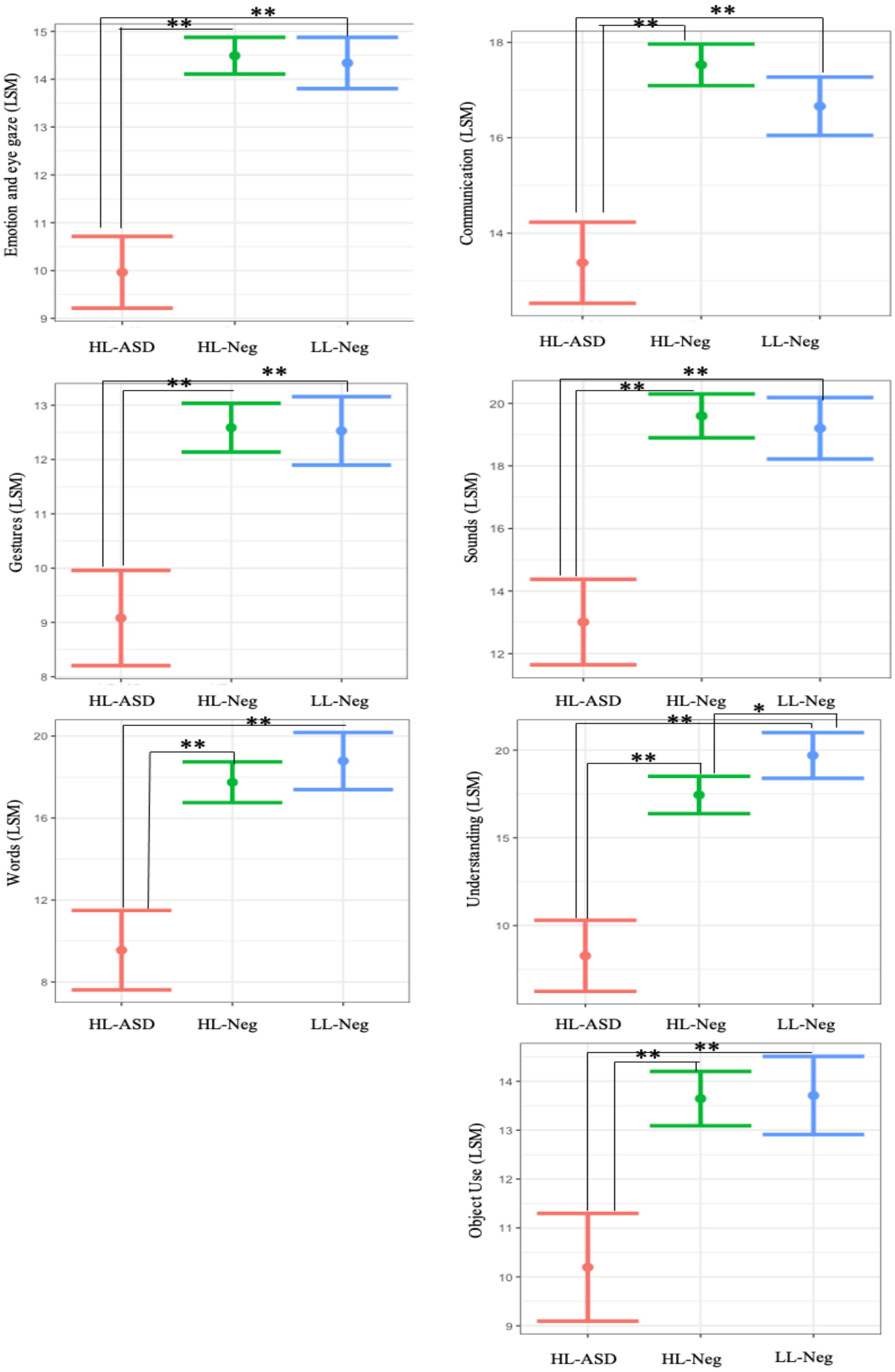

**p < .001; *p <. 05

Figure S1. *Least square means (LSM) of CSBS cluster scores by group at 24-months-of-ag*

Table S3. *Associations between social communication skills measured at 12-months and language abilities measured at 24-months in HL-ASD, HL-Neg, and LL-Neg infants.*

CSBS scores	HL-ASD			HL-Neg			LL-Neg		
	Estimate	*t*	*q value*	Estimate	*t*	*q value*	Estimate	*t*	*q value*
**MSEL Expressive Language**									
Emotion and Eye Gaze	_-.04	-.20	0.92	0.20	2.31	0.03*	0.21	1.65	0.10
Communication	0.15	0.91	0.92	0.15	2.46	0.02*	0.23	2.20	0.03*
Gestures	–0.13	–0.58	0.92	0.23	2.91	0.01*	0.36	3.29	<.01*
Sounds	-.02	-.09	0.92	0.16	2.79	0.01*	0.29	3.38	<.01*
Words	–.28	-.27	0.92	0.28	2.18	0.03*	0.56	2.43	0.02*
Understanding	0.87	1.24	0.92	0.28	2.82	0.01*	0.39	3.07	<.01*
Object use	–.05	–0.24	0.92	0.13	1.31	0.19	0.45	3.92	<.01*
**MSEL Receptive Language**									
Emotion and Eye Gaze	-0.06	-0.22	0.93	0.20	2.55	0.04*	0.01	0.96	0.92
Communication	0.17	0.82	0.93	0.11	2.21	0.06	0.03	0.42	0.79
Gestures	–0.07	–0.26	0.93	0.18	2.66	0.04*	0.13	1.69	0.33
Sounds	0.37	1.07	0.93	0.04	0.80	0.49	0.04	0.66	0.71
Words	-0.06	-0.46	0.93	0.02	0.21	0.83	0.23	1.48	0.33
Understanding	1.42	1.90	0.48	0.14	1.49	0.24	0.09	1.06	0.51
Object use	0.02	0.09	0.93	0.09	1.07	0.40	0.28	3.66	<.01*
**Vineland Expressive Language**									
Emotion and Eye Gaze	0.97	2.19	0.09	0.55	2.07	0.04*	0.64	1.86	0.15
Communication	0.04	0.13	0.89	0.84	4.59	<.01*	0.49	1.67	0.17
Gestures	0.32	0.69	0.69	0.75	3.23	<.01*	0.48	1.57	0.17
Sounds	0.45	0.91	0.64	0.68	3.96	<.01*	0.31	1.26	0.21
Words	4.27	2.09	0.10	0.99	2.51	0.02*	0.84	1.30	0.21
Understanding	2.05	2.55	0.10	0.50	1.62	0.11	1.02	2.86	0.04*
Object use	–0.08	-0.17	0.89	0.85	2.98	<.01*	0.80	2.47	0.05
**Vineland Receptive Language**									
Emotion and Eye Gaze	0.57	2.40	0.14	0.17	2.06	0.09	0.19	2.18	0.22

Communication	–0.02	–0.11	0.91	0.16	2.73	0.02*	0.03	0.41	0.80
Gestures	0.03	0.14	0.91	0.13	1.80	0.09	0.04	0.49	0.80
Sounds	0.22	0.84	0.27	0.09	1.76	0.09	0.01	0.25	0.80
Words	2.19	2.00	0.17	0.37	3.11	0.01*	0.07	0.44	0.80
Understanding	0.69	1.63	0.26	0.13	1.27	0.21	0.11	1.25	0.76
Object use	–0.09	–0.40	0.91	0.16	1.75	0.09	0.06	0.76	0.80

Table S4. *Associations between social communication skills measured at 24-months and language abilities measured at 36-months in HL-ASD, HL-Neg, and LL-Neg infants.*

CSBS scores	HL-ASD			HL-Neg			LL-Neg		
	Estimate	*t*	*q value*	Estimate	*t*	*q value*	Estimate	*t*	*q value*
**MSEL Expressive Language**									
Emotion and Eye Gaze	0.81	3.52	<.01*	–0.09	-0.48	0.63	–0.19	–0.63	0.63
Communication	0.45	1.86	0.10	0.29	2.17	0.05	0.10	0.36	0.72
Gestures	0.49	1.49	0.17	0.07	0.59	0.63	0.13	0.62	0.63
Sounds	0.49	3.20	<.01*	0.36	4.88	<.01*	0.29	2.17	0.13
Words	0.49	3.70	<.01*	0.27	4.79	<.01*	0.08	0.94	0.63
Understanding	0.44	3.26	<.01*	0.21	2.56	0.03*	0.43	2.36	0.13
Object use	–0.12	-0.40	0.69	0.23	2.08	0.06	–0.10	–0.62	0.63
**MSEL Receptive Language**									
Emotion and Eye Gaze	0.87	3.43	<.01*	-0.09	-0.50	0.61	–0.18	-0.55	0.94
Communication	0.48	1.79	0.11	0.19	1.47	0.25	0.01	0.03	0.97
Gestures	0.59	1.64	0.13	0.14	1.21	0.27	-0.01	–0.06	0.97
Sounds	0.49	2.94	<.01*	0.24	3.29	<.01*	0.18	1.12	0.97
Words	0.49	3.36	<.01*	0.20	3.63	<.01*	0.01	0.09	0.97
Understanding	0.59	3.99	<.01*	0.20	2.99	<.01*	0.54	2.73	0.07
Object use	0.01	0.05	0.96	0.13	1.20	0.27	0.03	0.14	0.97

Table S5. *General liner model exploring interaction effects of 12-month social communication skills by group on language abilities measured at 24-months*

CSBS scores	Interaction effect of CSBS scores*group			
	SS	*F*	*q* value	f^2
**MSEL EL**				
Emotion eye gaze (*N* =376)	2.3	0.07	0.93	0
Communication (*N* =393)	23.9	0.70	0.86	0
Gestures (*N* =393)	73.8	2.19	0.40	0.01
Sounds (*N* =393)	16.0	0.48	0.86	0
Words (*N* =393)	24.5	0.71	0.86	0
Understanding (*N* =260)	5.2	0.16	0.93	0
Object use (*N* =332)	77.3	2.39	0.40	0.02
**Vineland EL**				
Emotion eye gaze (*N* =368)	199	0.70	0.58	0
Communication (*N* =384)	809	2.94	0.38	0.02
Gestures (*N* =384)	306	1.08	0.58	0
Sounds (*N* =384)	91	0.33	0.72	0
Words (*N* =396)	356	1.26	0.58	0
Understanding (*N* =253)	392	1.48	0.58	0.01
Object use (*N* =325)	228	0.82	0.58	0
**MSEL RL**				
Emotion eye gaze (*N* =376)	19.2	0.64	0.79	0
Communication (*N* =393)	1.6	0.05	0.94	0
Gestures (*N* =393)	8.7	0.29	0.87	0
Sounds (*N* =393)	38.8	1.31	0.79	0
Words (*N* =393)	16.9	0.57	0.79	0
Understanding (*N* =260)	81.4	2.46	0.61	0.02
Object use (*N* =332)	24.7	0.80	0.79	0
**Vineland RL**				
Emotion eye gaze (*N* =368)	153	4.84	0.03*	0.03
Communication (*N* =384)	37.3	1.16	0.55	0.08
Gestures (*N* =383)	16.0	0.49	0.71	0.06
Sounds (*N* =384)	108.1	3.40	0.08	0.02
Words (*N* =384)	145.1	4.65	0.03*	0.06
Understanding (*N* =253)	18.8	0.57	0.71	0.03
Object use (*N* =325)	3.8	0.12	0.89	0

Note: The degree of freedom for all models was 2.

Table S6. *General liner model exploring interaction effects of 24-month social communication skills by group on language abilities measured at 36-months*

CSBS scores	Interaction effect of CSBS scores*group			
	SS	*F*	*q* value	f^2
**MSEL EL**				
Emotion eye gaze (*N* =194)	364.9	9.24	<.01*	0.10
Communication (*N* =200)	34.4	0.82	0.44	0
Gestures (*N* =200)	78.5	1.80	0.37	0.02
Sounds (*N* =200)	39.8	1.13	0.38	0.01
Words (*N* =200)	99.7	2.78	0.22	0.03
Understanding (*N* =144)	55.16	1.34	0.37	0.02
Object use (*N* =192)	59.1	1.36	0.37	0.09
**MSEL RL**				
Emotion eye gaze (*N* =194)	350	8.89	<.01*	0.10
Communication (*N* =200)	51.1	1.19	0.36	0.01
Gestures (*N* =200)	56.2	1.29	0.36	0.01
Sounds (*N* =200)	76.2	1.97	0.25	0.02
Words (*N* =200)	377	14.80	0.05	0.07
Understanding (*N* =144)	195.4	3.81	0.04*	0.06
Object use (*N* =192)	17.8	0.41	0.66	0

Note: The degree of freedom for all models was 2.
